# Injustice Experience Questionnaire, Japanese Version: Cross-Cultural Factor-Structure Comparison and Demographics Associated with Perceived Injustice

**DOI:** 10.1371/journal.pone.0160567

**Published:** 2016-08-03

**Authors:** Keiko Yamada, Tomonori Adachi, Akira Mibu, Tomohiko Nishigami, Yasushi Motoyama, Hironobu Uematsu, Yoichi Matsuda, Hitoaki Sato, Kenichi Hayashi, Renzhe Cui, Yumiko Takao, Masahiko Shibata, Hiroyasu Iso

**Affiliations:** 1 Public Health, Department of Social Medicine, Osaka University Graduate School of Medicine, Suita-shi, Osaka, Japan; 2 Center for Pain Management, Osaka University Hospital, Suita-shi, Osaka, Japan; 3 Department of Pain Medicine, Osaka University Graduate School of Medicine, Suita-shi, Osaka, Japan; 4 Department of Rehabilitation, Tanabe Orthopedics, Osaka, Japan; 5 Department of Nursing and Physical Therapy, Konan Women’s University, Kobe, Japan; 6 Division of Anesthesiology, Kobe University Graduate School of Medicine, Kobe, Japan; 7 Department of Anesthesiology & Intensive Care Medicine, Osaka University Graduate School of Medicine, Suita-shi, Osaka, Japan; 8 Department of Mathematics, Keio University, Yokohama, Japan; Hokkaido University, JAPAN

## Abstract

**Objective:**

The Injustice Experience Questionnaire (IEQ) assesses injury-related perceived injustice. This study aimed to (1) develop a Japanese version (IEQ-J), (2) examine its factor structure, validity, and reliability, and (3) discover which demographic variable(s) positively contributed to prediction of IEQ-J scores.

**Methods:**

Data from 71 patients (33 male, 38 female; age = 20+) with injury pain were employed to investigate factor structure by exploratory and confirmatory factor analyses. Concurrent validity was examined by Pearson correlation coefficients among the IEQ-J, Brief Pain Inventory (BPI), and Pain Catastrophizing Scale (PCS). Internal consistency was investigated by Cronbach’s alpha, and test-retest reliability was indicated with intra-class correlations (ICCs) in 42 of 71 patients within four weeks. Relations between demographic variables and IEQ-J scores were examined by covariance analysis and linear regression models.

**Results:**

IEQ-J factor structure differed from the original two-factor model. A three-factor model with *Severity/irreparability*, *Blame/unfairness*, and *Perceived lack of empathy* was extracted. The three-factor model showed goodness-of-fit with the data and sufficient reliability (Cronbach’s alpha of 0.90 for total IEQ-J; ICCs = 0.96). Pearson correlation coefficients among IEQ-J, BPI, and PCS ranged from 0.38 to 0.73. Pain duration over a year (regression coefficient, 11.92, 95%CI; 5.95–17.89) and liability for injury on another (regression coefficient, 12.17, 95%CI; 6.38–17.96) predicted IEQ-J total scores.

**Conclusions:**

This study evidenced the IEQ-J’s sound psychometric properties. The three-factor model was the latter distinctive in the Japanese version. Pain duration over a year and injury liability by another statistically significantly increased IEQ-J scores.

## Introduction

The Injustice Experience Questionnaire (IEQ) is an assessment tool for measuring injury-related perceived injustice [1]. Relationships between perception of injustice and health-related issues have long been discussed. For example, associations between injustice and sleeping problems [2], cerebrovascular disease [3], and sick leave from work [4] have been reported. Victims injured by another’s error or negligence (e.g., whiplash injury) are likely to experience injustice that prevents them from recovering and returning to work [5]. Perceived injustice might predictively indicate prognosis and be an important therapeutic target for recovering from severe injury [5]. Thus, perception of injustice should be investigated as a negative belief among traumatized patients.

Furthermore, injured patients with chronic pain not only perceive injustice, but also catastrophize their experience of pain [1]. Total scores of the Pain Catastrophizing Scale (PCS), a measure of exaggerating pain among chronic patients, were highly correlated with total scores of the IEQ [1]. Pain catastrophizing is also an exaggerated negative belief that prevents patients from recovering and seems to be a treatment target for patients suffering chronic pain [6]. The original version of the PCS was in English, but has already been translated into many languages; for instance, Brazilian, Portuguese [7], Chinese [8], Italian [9], Korean [10], Turkish [11], and Japanese [12]. Moreover, cross-cultural assessment of the PCS has been implemented [7–12].

The IEQ’s original version was developed in a Canadian sample by Sullivan et al. in English and French [1], and the Spanish version was used in a previous study of patients with fibromyalgia, although fibromyalgia is not injury-related pain [13]. The IEQ has never been translated into Japanese. Therefore, we developed the Japanese version of the IEQ (IEQ-J) to examine perceived injustice in the Japanese clinical population.

The original IEQ version had two factors, *Severity/irreparability* and *Blame/unfairness*, in the Canadian population [1], and its factor structure has also already been confirmed in an Australian compensable population [14]. However, the IEQ’s factor structures might differ in Canadian, Australian, and Japanese populations. Thus, we re-examined its factor structure.

Furthermore, we researched the association between demographic variables regarding injury-related pain and IEQ-J scores to reinforce evidence from previous studies [1,13,15]. The following demographic variables were examined: duration of pain, cause of injury, liability for injury, employment status, compensation, and dispute. Presumably, victims injured by another’s error or negligence and the injured who are compensated are more likely to perceive injustice than persons with self-inflicted injury or without compensation. Sullivan et al. indicated that motor vehicle accidents were associated with higher IEQ scores [1]. Ferrari reported that whiplash victims at 6-months post-injury showed higher IEQ scores than victims at 3-months post-injury. Higher IEQ scores were also a risk factor for lack of recovery [15].

The present study’s three objectives were to develop the Japanese version of the IEQ, to examine its factor structure, validity, and reliability, and to discover which demographic variable(s) positively contributed to prediction of IEQ-J scores.

## Materials and Methods

### Translation of the IEQ into Japanese

**Fig 1:**
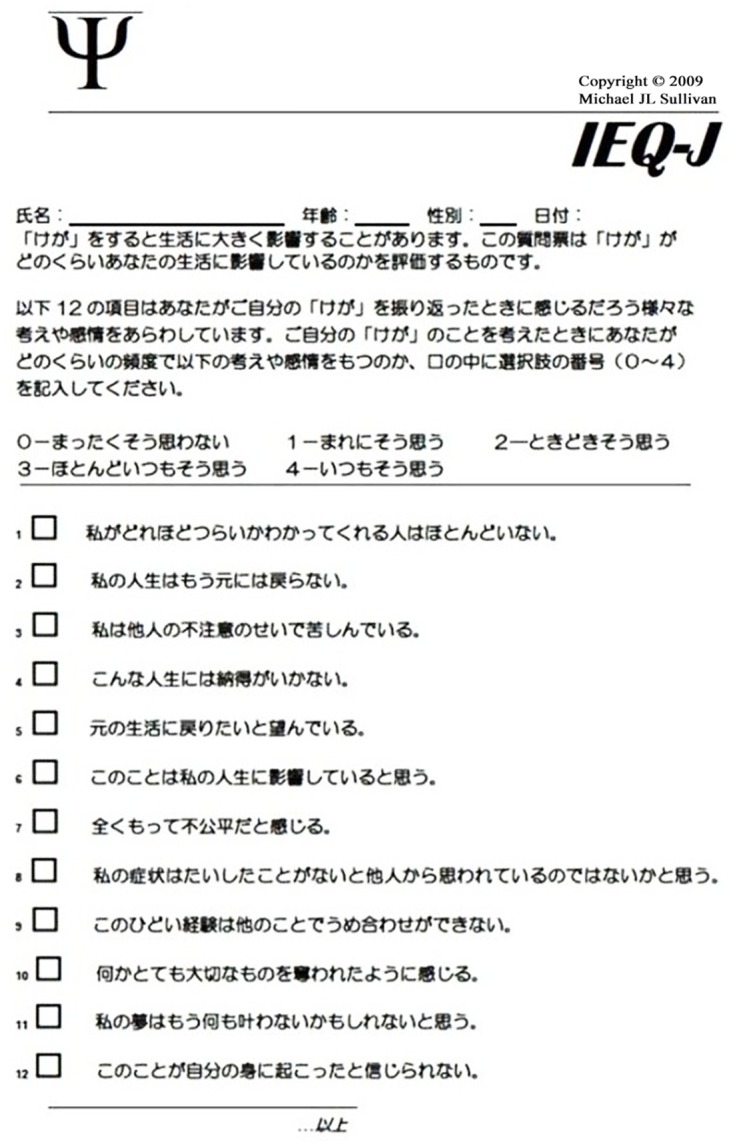
The Japanese form of the IEQ-J.

Language and cultural equivalence should be adapted from an original to a translated questionnaire [16]. Therefore, the IEQ’s translation into Japanese was based on guidelines for cross-cultural adaptation of self-report measures [17]. After obtaining authorization to develop the Japanese version from the original IEQ’s author (M.S.), the original was translated into Japanese by four persons: a medical doctor (K.Y.), a physical therapist (T.N.), and a clinical psychologist (T.A.)—all native Japanese speakers—and a medical student (D.W.) who is a native English speaker also speaking Japanese. This initial translation was then reverse translated to English by another native English speaker (E.S.), who is bilingual in English and Japanese and who had no prior knowledge of the original IEQ. This back-translated version was compared with the original version and judged for translation clarity and linguistic equivalence by the four persons noted above, another medical doctor (M.S.), the corresponding author (H.I.), and the original IEQ’s author (M.S.). Consequently, minor modifications were made to the initial Japanese translation. Then, this final questionnaire was named the Japanese version of the IEQ (IEQ-J). The Japanese form of the IEQ-J was shown in Fig 1. The original author of IEQ (M.S.) was approved the use of this Japanese form for free.

### Sample Population

During the current study from December 2014 to May 2015, 74 patients with pain owing to injury participated. They answered three questionnaires and provided their background information. Two participants with acute pain within a month of injury and a participant who did not respond to the IEQ-J were excluded. Thus, analysis included 71 participants (33 male, 38 female), aged 20 and older, from four facilities in Japan—31 and 24 participants from two university hospitals, two participants from a prefectural hospital, and 14 participants from an orthopedic clinic. There is no single method for factor analysis of calculating a minimum sample size [18]. Many recommend a subject-to-variables ratio of 5 to 10:1, with a minimum of 50 or 100 samples [18,19]. In the current study, the subject-to-variables ratio was 5.9:1, with over 50 subjects. The sample size of the current study thus exceeded minimum requirements.

### Measures

#### Perceived Injustice

Developed by Sullivan et al., the Injustice Experience Questionnaire (IEQ) is a 12-item, self-report scale for measuring perceived injustice associated with injury [1]. Responders rate the frequency with which they have experienced each of 12 pain-related perceptions. The IEQ uses a 5-point Likert-type scale, ranging from 0 (never) to 4 (all the time) [1]. The original IEQ’s items are as follows:

- Item 1. Most people don’t understand how severe my condition is.
- Item 2. My life will never be the same.
- Item 3. I am suffering because of someone else’s negligence.
- Item 4. No one should have to live this way.
- Item 5. I just want to have my life back.
- Item 6. I feel that this has affected me in a permanent way.
- Item 7. It all seems so unfair.
- Item 8. I worry that my condition is not being taken seriously.
- Item 9. Nothing will ever make up for all that I have gone through.
- Item 10. I feel as if I have been robbed of something very precious.
- Item 11. I am troubled by fears that I may never achieve my dreams.
- Item 12. I can’t believe this has happened to me [1].

A two-factor model was proposed for the IEQ’s original version: *Severity/irreparability* comprised Items 1, 2, 4, 5, 6, and 8; *Blame/unfairness* comprised Items 3, 7, 9, 10, 11, and 12 [1]. Cronbach’s alpha for the original IEQ was 0.92.

#### Pain intensity and interference

The Brief Pain Inventory (BPI) was originally developed to assess the intensity and interference of cancer-related pain. It consists of a mannequin for describing pain sites and two aspects for evaluation: pain intensity and pain interference [20]. The Japanese version of the BPI (BPI-J) was developed by Uki et al. [21]. Cronbach’s alpha for the BPI-J’s pain intensity and pain interference scale were both 0.81 [21]. The BPI’s pain intensity scale is equal to the numerical rating scale (NRS); it is supported by the initiative on methods, measurement, and pain assessment in clinical trials (IMMPACT) [22]. The NRS assesses pain severity at its “worst,” “least,” and “average” for the last 24 hours and “now” on a 0 (= no pain) to 10 (= worst pain imaginable) scale. On the BPI’s pain interference scale, responders answer how their pain interferes with seven daily activities; general activity, mood, walking, work, relations with others, sleep, and enjoyment of life, from 0 (= does not interfere) to 10 (= interferes completely) [20,22].

The original version, the McGill Pain Questionnaire (MPQ) [23] was used to measure pain intensity, and the Pain Disability Index (PDI) [24] was used to measure pain interference. Because there was no Japanese version of the PDI, we used the BPI to assess both pain intensity and interference.

#### Pain Catastrophizing

The Pain Catastrophizing Scale (PCS) consists of 13 items that describe individuals’ specific beliefs about their pain and evaluates catastrophic thinking about pain [6]. Each item is rated on a 5-point Likert-type scale ranging from 0 (not at all) to 4 (all the time); a PCS total score is calculated by summing the 13 items from 0 to 52 points. The PCS has three subscales to assess Helplessness, Magnification, and Rumination. The Japanese PCS version, including the two subscales of Helplessness and Rumination, has been assessed for validity and reliability [12]. A previous study indicated that the PCS showed high internal consistency (Cronbach’s alpha = 0.87) and a strong correlation between the IEQ and the PCS (coefficient of correlation = 0.75, *p* < 0.01) [1].

#### Demographic variables

In addition to these previous three measures, participants were asked about their backgrounds: age, sex, duration of pain, cause of injury, liability of injury, employment status, compensation, and dispute. Duration of pain was divided into six categories: under a month, from a month to under three months, from three months to under six months, from six months to under a year, from a year to under five years, and over five years. Cause of injury included four categories: traffic accidents, workers’ accidents (except traffic accidents), falls, and others. There were four choices of liability for injury: self-inflicted, another person, both, or unsure. Employment status was divided into three categories: on sick leave, working, or non-employed. Compensated and under dispute were yes-or-no questions.

### Statistical Analysis

#### Validity and Reliability

First, the IEQ-J’s structural validity was supported by exploratory factor analysis (EFA) and confirmatory factor analysis (CFA). EFA was performed to determine the IEQ-J’s factor structure using promax rotation and the maximum likelihood estimation method. Correlations between IEQ-J factors were also calculated. CFA was enforced to confirm the factor structure derived from EFA by accounting for variation and covariation among the 12 items and using fit indexes for three different factor structure models (Models 1–3). Model 1 had one factor; model 2 had two factors, consistent with the original IEQ version [1]; and the current study’s EFA proposed three factors. Fit indices were selected by reference to a previous paper, which reported CFAs of the IEQ and PCS [14] and Guidelines for Determining Model Fit [25]. As absolute fit indices, the χ^2^, χ^2^/df, the root mean square error of approximation (RMSEA), and the standardized root mean square residual (SRMR) were used. According to values of the RMSEA, <0.05 suggests a good fit, from 0.08 to 0.10 indicates a moderate fit, and >0.10 means a poor fit [25]. Values for SRMR in the range of 0.09 or lower indicate a good fit [25]. On behalf of incremental fit indices, the comparative fit index (CFI) and the Tucker-Lewis Index (TLI) were used [25]. A cut-off close to 0.95 of the CFI and the TLI is recommended for relatively good fit, as indicated by Hu and Bentler [26].

Second, the IEQ-J’s concurrent validity was calculated by Pearson’s correlation coefficients among the BPI, the PCS, and the IEQ-J.

Third, internal consistency supported by Cronbach’s alpha and test-retest reliability also demonstrated the IEQ-J’s reliability. Intra-class correlations (ICCs) were computed to evaluate test-retest reliability. The sample size for ICC analysis was determined with the following assumptions: the null hypothesis H_0_ is that the ICC is 0.60, the alternative hypothesis H_1_ is that the ICC is 0.80. When the power is 0.90, the minimum required size of the sample is 39. In the present study, 42 of 71 participants who had returned to the clinic within one to four weeks of first completing the IEQ-J questionnaire, completed the secondary IEQ-J questionnaire. We then performed ICC analyses for the IEQ-J’s primary and secondary total scores.

### Further analyses

To discover what elements increased perceived injustice and pain catastrophizing, linear regression models were created for associations between demographic variables and the IEQ-J total score, the IEQ-J subscale scores, and the total PCS score.

The significance level of statistical hypothesis testing was set at *p* = 0.05. CFA was performed using IBM SPSS Amos ver23 (IBM Corp., New York, USA); sample size for ICCs analysis was determined by PASS software ver13 (NCSS, Utah, USA), and ICCs analysis used IBM SPSS ver21 (IBM Corp., New York, USA). Other than those listed, statistical analyses were performed using SAS version 9.4 (SAS Institute Inc., North Carolina, USA).

### Ethical Provisions

All procedures followed were in accordance with the ethical standards of the responsible committee on human experimentation, and with the Helsinki Declaration of 1975, as revised in 2000 [27]. This study was approved by the Osaka University Hospital Institutional Review Boards (No. 14248) and Kobe University Graduate School of Medicine Institutional Review Boards (No. 1703). Written informed consent was obtained from all patients included in the study.

## Results

### Sample characteristics

Demographic valuables are shown in Table 1. The mean age of the 71 participants was 50.7 (SD 14.4). The number of males was 33 and females, 38 (46.5% and 53.5%, respectively). Ratio of participants’ duration of pain was as follows: from one month to less than three months (5.6%); from three months to less than six months (4.2%); from six months to less than a year (12.7%); from a year to less than five years (32.4%); and five years or more (45.1%). Proportions of pain sites were head, face, and mouth (16.9%); cervical region (23.9%); upper shoulder and upper limbs (63.4%); thoracic region (8.5%); abdominal region (7.0%); low back, lumbar spine, sacrum, and coccyx (35.2%); lower limbs (46.5%); pelvic region (21.1%); and anal, perineal, and genital region (0%). Widespread pain, at more than three major sites, was (35.2%). Causes of participants’ pain included traffic accidents (52.1%), worker’s accidents (except for traffic accidents) (12.7%), falls (16.9%), and others (18.3%). Subjective judgments about liability of injury included one’s self (25.4%), another person (53.5%), both one’s self and another (15.5%), and not sure (5.6%). Participants’ employment statuses included on sick leave (39.4%), working (38.0%), and unemployed (22.5%). Among the 71 participants, 61.4% were compensated, 38.6% were uncompensated, and one participant did not respond to this question. The number of cases under dispute was 15 (21.7%), not under dispute 52 (78.3%), and the number of non-responders was two of 71 participants.

**Table 1:** Sample characteristics.

	Frequency (n)	%
Age (years)	50.7±14.4	
Number of subjects	71	
Number of women, n (%)	38	53.5
Facility		
A university hospital	31	43.7
B university hospital	24	33.8
C prefectural hospital	2	2.8
D orthopedic clinic	14	19.7
Duration of pain		
≥ 1 month, < 3 months	4	5.6
≥ 3 months, < 6 months	3	4.2
≥ 6 months, < 1year	9	12.7
≥ 1 year, < 5years	23	32.4
≥ 5 years	32	45.1
Pain site		
Head, face, and mouth	12	16.9
Cervical region	17	23.9
Upper shoulder and upper limbs	45	63.4
Thoracic region	6	8.5
Abdominal region	5	7.0
Low back, lumbar spine, sacrum, and coccyx	25	35.2
Lower limbs	33	46.5
Pelvic region	15	21.1
Anal, perineal, and genital region	0	0.0
More than three major sites	25	35.2
Cause of pain		
Traffic accidents	37	52.1
Workers’ accidents (except traffic accidents)	9	12.7
Falls	12	16.9
Others	13	18.3
Liability for injury		
Self-inflicted	18	25.4
Another person	38	53.5
Both	11	15.5
Not sure	4	5.6
Employment status		
On sick leave	28	39.4
Working	27	38.0
Non-employed	16	22.5
Compensation		
Compensated	43	61.4
Uncompensated	27	38.6
Non-Responder	1	-
Dispute		
Under dispute	15	21.7
Not disputed	54	78.3
Non-Responder	2	-

Note. Age: Mean ±SD

### Factor structure

#### Exploratory factor analysis

Results of EFA for the IEQ-J are shown in Table 2, and correlations between factors are indicated in Table 3. In IEQ-J, three factors were extracted: *Severity/irreparability* consisted of Items 2, 5, 6, 9, 10, and 11; *Blame/unfairness* consisted of Items 3, 4, 7, and 12; and *Perceived lack of empathy* included Items 1 and 8. Correlation coefficients between IEQ-J factors ranged from 0.48 to 0.56, indicating moderate correlation.

**Table 2:** Factor loadings and internal consistency of the IEQ-J for the three factors.

Item	Cronbach’s alpha of IEQ-J total score = 0.90			Item/total correlation (r)
	Severity/irreparability	Blame/unfairness	Perceived lack of empathy	
	(α = 0.89)	(α = 0.79)	(α = 0.74)	
Item 2 My life will never be the same.	**0.64**	0.02	0.21	0.71
Item 5 I just want to have my life back.	**0.42**	0.33	-0.07	0.58
Item 6 I feel that this has affected me in a permanent way.	**0.63**	0.22	-0.07	0.67
Item 9 Nothing will ever make up for all that I have gone through.	**0.71**	0.01	0.18	0.75
Item 10 I feel as if I have been robbed of something very precious.	**0.99**	-0.04	-0.06	0.76
Item 11 I am troubled by fears that I may never achieve my dreams.	**0.82**	-0.11	0.04	0.64
Item 3 I am suffering because of someone else's negligence.	-0.10	**0.81**	0.04	0.55
Item 4 No one should have to live this way.	**0.43**	**0.58**	-0.04	0.79
Item 7 It all seems so unfair.	-0.01	**0.79**	0.02	0.58
Item 12 I can’t believe this has happened to me.	0.04	**0.41**	0.04	0.40
Item 1 Most people don’t understand how severe my condition is.	0.15	0.23	**0.56**	0.68
Item 8 I worry that my condition is not being taken seriously.	0.01	-0.03	**0.76**	0.48

Note. Factor loadings greater than 0.40 are in bold

IEQ-J; Japanese version of the Injustice Experience Questionnaire

**Table 3:** Correlations between factors of IEQ-J.

Factor	Factor 1	Factor 2	Factor 3
Factor 1: Severity/irreparability	1.00	0.55	0.56
Factor 2: Blame/unfairness	0.55	1.00	0.48
Factor 3: Perceived lack of empathy	0.56	0.48	1.00

Note. IEQ-J; Japanese version of the Injustice Experience Questionnaire

#### Confirmatory factor analysis

**Fig 2:**
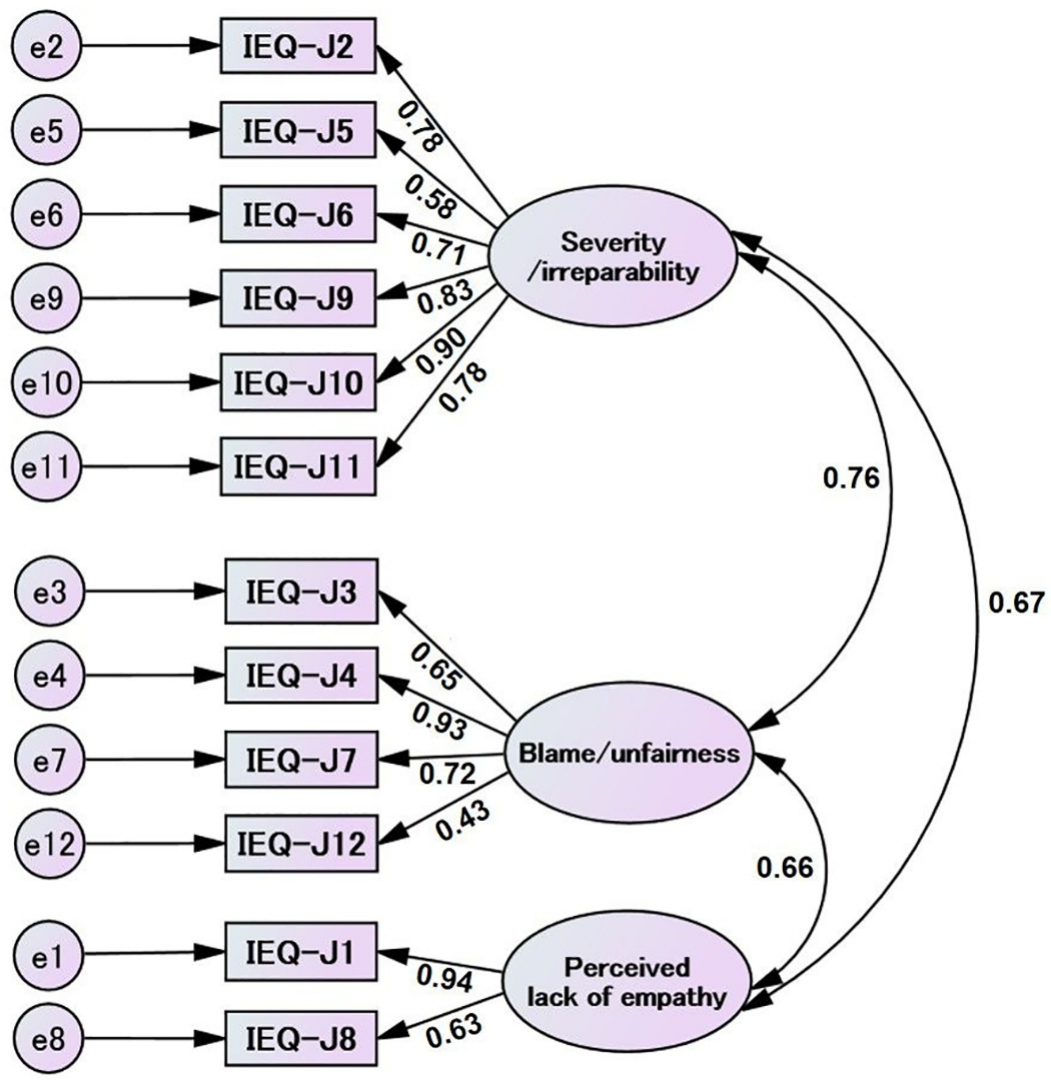
Three-factors model of the IEQ-J. Three-factors model of the IEQ-J, and Error terms (e1–e12) are also indicated. Note. IEQ-J, Japanese version of Injustice Experience Questionnaire

In Fig 2, the three-factor model of the IEQ-J, derived by EFA, is shown with error terms e1–e12, and standardized parameter estimates ranging from 0.43 to 0.94.

In Table 4, a summary of goodness-of-fit indices for the three models is indicated. In model 1, although 0.085 of SRMRs (0.09 or lower) were a good fit [25], RMSEA was 0.13 (>0.10), and the CFI and TLI were under 0.95, indicating poor fit [26]. In model 2, 0.085 of SRMRs ≤ 0.09 was a good fit, but RMSEA was 0.13 (>0.10), and the CFI and TLI of model 2 were 0.83 (around 0.95), indicating a poor fit [26]. RMSEA of model 3 was 0.06 (<0.10), a good fit for the IEQ-J [25]. Whereas the three models’ SRMRs range was 0.09 or lower, indicating a good fit [26], the SRMR of model 3 (0.063), was better than that of model 1 (0.085), and of model 2 (0.084). Furthermore, the CFI (0.98) and TLI of Model 3 (0.97) were recognized as a good fit (around 0.95) [26].

**Table 4:** Summary of goodness-of-fit indices for the three models of the IEQ-J.

	χ^2^ (df)	χ^2^/df	RMSEA (90%CI)	SRMR	CFI	TLI
Model 1: one factor (12 items)	113.23	2.10	0.13 (0.09–0.16)	0.085	0.86	0.83
Model 2: original two factors (6 items + 6 items)	112.98	2.13	0.13 (0.09–0.16)	0.084	0.86	0.83
Model 3: three factors (6 items + 4 items + 2 items)	61.76	1.21	0.06 (0.00–0.10)	0.063	0.98	0.97

Note. *Χ*^*2*^, chi square; *df*, degrees of freedom; RMSEA, root mean square error of approximation; CI, confidence intervals; SRMR, standardized root mean square residual; CFI, comparative fit indices; TLI, Tucker-Lewis Index

#### Concurrent validity

Correlation coefficients of the IEQ-J total score and total/subscales scores of the BPI ranged from r = 0.38 to 0.68, *p* < 0.01 (Table 5). Correlation coefficients of the original IEQ total score and the McGill Pain Questionnaire score were r = 0.54, *p* < 0.01, and the Pain Disability Index score was r = 0.44, r < 0.01 [1]. Concurrent validity of the IEQ-J was observed in this study population.

**Table 5:** Correlation coefficients (95%CI) among IEQ-J, PCS, and BPI.

Variable	1	2	3	4	5	6
1 IEQ-J Total						
2 PCS Total	0.73 (0.59–0.82)^‡^					
3 BPI Average pain intensity	0.55 (0.36–0.69)^‡^	0.53 (0.34–0.68)^‡^				
4 BPI Maximum pain	0.46 (0.25–0.62)^‡^	0.43 (0.21–0.60)^‡^	0.81 (0.71–0.88)^‡^			
5 BPI Minimum pain	0.38 (0.16–0.56)^†^	0.31 (0.08–0.51)^†^	0.63 (0.46–0.75)^‡^	0.58 (0.40–0.71)^‡^		
6 BPI Now	0.55 (0.36–0.69)^‡^	0.56 (0.37–0.70)^‡^	0.86 (0.78–0.91)^‡^	0.78 (0.66–0.85)^‡^	0.58 (0.39–0.71)^‡^	
7 BPI Interference scale	0.68 (0.52–0.79)^‡^	0.70 (0.55–0.80)^‡^	0.69 (0.54–0.79)^‡^	0.61 (0.43–0.74)^‡^	0.43 (0.21–0.60)^‡^	0.74 (0.60–0.83)^‡^

Note: n = 71;

^†^ p <0.01,

^‡^ p <0.001

IEQ-J, Japanese version of the Injustice Experience Questionnaire; PCS, Pain Catastrophizing Scale; BPI, Brief Pain Inventory

#### Reliability

Cronbach’s alpha of the IEQ-J’s total score was 0.90; of *Severity/irreparability*, 0.89; of *Blame/unfairness*, 0.79, and of *Perceived lack of empathy*, 0.74 (Table 2). ICCs of the IEQ-J total and subscale scores ranged from 0.93 to 0.96. Over 0.70 of ICC was assessed a reproducible result (Table 6) [28].

**Table 6:** Intraclass correlation coefficient of Japanese version of the Injustice Experience Questionnaire.

Items, n = 42		Test-retest ICC
IEQ-J Total	Item 1–Item 12	0.96 (0.93–0.98)
IEQ-J Severity/irreparability	Item 2, Item 5, Item 6, Items 9–11	0.95 (0.90–0.97)
IEQ-J Blame/unfairness	Item 3, Item 4, Item 7, Item 12	0.93 (0.87–0.96)
IEQ-J Perceived lack of empathy	Item 1, Item 8	0.96 (0.93–0.98)

Note. ICC, intraclass correlation coefficient; IEQ-J, Japanese version of the Injustice Experience Questionnaire

#### Demographic variables related to injury or pain

Associations between demographic valuables and mean value of IEQ-J total scores are indicated in Table 7. The mean value of the IEQ-J total score was 24.1 (12.0 SD), and the median value was 25.0. The mean value of IEQ-J total scores in participants whose duration of pain was over a year (26.4) was higher than that in participants whose duration of pain was under a year (16.1). Participants who believed liability for injury rested with another person (27.7) also experienced pain for longer than participants who believed liability lay with themselves or with both parties, or who were not sure (20.0). According to cause of injury, employment status, compensation, and dispute, IEQ-J total scores’ mean value of participants whose conditions were considered to have increased IEQ-J scores. Scores of participants injured by traffic accidents, on sick leave, compensated, and injury under dispute were not significantly higher than that of participants with workers’ accidents, falls, or other; working or non-employed; uncompensated or non-responder; and not disputed or non-responder.

**Table 7:** Mean values of IEQ-J total scores according to sample characteristics.

Variables (n = 71)	n	*Mean (SE)*
Duration of pain		
< 1 year	16	16.1 (2.8)
≥ 1 year	55	26.4 (1.5)^†^
Cause of injury		
Workers’ accidents, falls, or others	34	24.6 (2.1)
Traffic accidents	37	23.6 (2.0)
Liability for injury		
Self-inflicted, both, or not sure	33	20.0 (2.0)
Another person	38	27.7 (1.9)^†^
Employment status		
Working or non-employed	43	22.8 (1.8)
On sick leave	28	26.1 (2.3)
Compensation		
Uncompensated or non-responder	43	21.6 (2.3)
Compensated	28	25.7 (1.8)
Dispute		
Not disputed or non-responder	56	23.1 (1.6)
Under dispute	15	27.8 (3.1)

Note. Analysis of covariance was used to test for differences from the category of <1 year, workers’ accidents, falls, or others; both or not sure; working or non-employed; uncompensated or non-responder; and not disputed or non-responder. SE, standard errors:

^†^p< 0.01.

Results of multiple/simple regression analyses to examine associations between demographic variables and IEQ-J total and subscale scores and PCS total scores are shown in Table 8. Pain duration over a year contributed significant variance to higher IEQ-J total scores (regression coefficient (B) of multiple regression = 11.92, 95%CI: 5.95–17.89, B of single regression = 10.3, 95%CI: 3.75–16.69); subscales (*Severity/irreparability*, B of multiple regression = 7.16, 95%CI: 3.65–10.67, B of single regression = 6.59, 95%CI: 2.92–10.27; *Blame/unfairness*, B of multiple regression = 3.13, 95%CI: 0.85–5.43, B of single regression = 2.18, 95%CI: -0.41–4.77; *Perceived lack of empathy*, B of multiple regression = 1.62, 95%CI: 0.28–2.96, B of single regression = 1.50, 95%CI: 0.24–2.77). Liability for injury by another person also contributed significant variance to higher IEQ-J total scores (B of multiple regression = 12.17, 95%CI: 6.38–17.96, B of single regression = 7.69, 95%CI: 2.24–13.14) and subscales (*Severity/irreparability*, B of multiple regression = 5.19, 95%CI: 1.79–8.59, B of single regression = 2.69, 95%CI: -0.60–5.98; *Blame/unfairness*, B of multiple regression = 5.71, 95%CI: 3.49–7.93, B of single regression = 4.30, 95%CI: 2.34–6.26). Moreover, compensation contributed significant variance to higher IEQ-J scores for *Severity/irreparability* (B of multiple regression = 3.24, 95%CI: 0.06–6.41, B of single regression = 3.40, 95%CI: 0.08–6.73). Traffic accident injury seems to be an independent factor for decreasing IEQ-J total score on multiple regression (B = -6.27, 95%CI: -11.81– -0.74); *Severity/irreparability* (B = -3.28, 95%CI: -6.53– -0.03); and *Blame/unfairness* (B = -2.32, 95%CI: -4.45–0.20). However, traffic accident injury did not significantly increase IEQ-J scores in simple regression analysis. Pain duration over a year and compensation contributed significant variance to higher PCS total scores; pain duration over a year, B of multiple regression = 10.41, 95%CI: 4.22–16.60, B of single regression = 9.40, 95%CI: 3.21–15.59; and compensated, B of multiple regression = 6.30, 95%CI: 0.71–11.89, B of single regression = 7.20, 95%CI: 1.82–12.58. Liability for injury of another did not significantly increase the total PCS score.

**Table 8:** Multiple or simple regression analysis examining associations between sample characteristics and perceived injustice or pain catastrophizing.

	Multiple regression analysis	Simple regression analysis
	Regression coefficient (95%CI)	Regression coefficient (95%CI)
**Dependent = IEQ-J Total**	**(R**^**2**^ **= 0.29)**	
Age	0.11 (-0.09–0.31)	-0.05 (-0.25–0.15)
Sex: women	0.71 (-4.55–5.96)	0.16 (-5.60–5.91)
Duration: ≥ 1 year	11.92 (5.95–17.89)	10.3 (3.75–16.69)
Cause of injury: traffic accident	-6.27 (-11.81– -0.74)	-0.97 (-6.71–4.77)
Liability for injury: another person	12.17 (6.38–17.96)	7.69 (2.24–13.14)
Employment status: on sick leave	2.79 (-2.29–7.88)	3.34 (-2.48–9.16)
Compensated	4.76 (-0.66–10.17)	4.15 (-1.64–9.94)
Under dispute	1.56 (-5.18–8.30)	4.71 (-2.23–11.65)
**Dependent = IEQ-J Severity/irreparability**	**(R**^**2**^ **= 0.28)**	
Age	0.09 (-0.03–0.21)	-0.02 (-0.14–0.10)
Sex: women	-0.76 (-3.85–2.32)	-1.38 (-4.72–1.95)
Duration: ≥ 1 year	7.16 (3.65–10.67)	6.59 (2.92–10.27)
Cause of injury: traffic accident	-3.28 (-6.53– -0.03)	-0.09 (-4.53– -2.14)
Liability for injury: another person	5.19 (1.79–8.59)	2.69 (-0.60–5.98)
Employment status: on sick leave	1.73 (-1.25–4.72)	2.08 (-1.31–5.46)
Compensated	3.24 (0.06–6.41)	3.40 (0.08–6.73)
Under dispute	2.59 (-1.37–6.54)	3.50 (-0.50–7.51)
**Dependent = IEQ-J Blame/unfairness**	**(R**^**2**^ **= 0.30)**	
Age	0.02 (-0.05–0.09)	-0.02 (-0.10–0.06)
Sex: women	1.50 (-0.51–3.52)	1.64 (-0.54–3.82)
Duration: ≥ 1 year	3.13 (0.85–5.43)	2.18 (-0.41–4.77)
Cause of injury: traffic accident	-2.32 (-4.45–0.20)	0.34 (-1.87–2.55)
Liability for injury: another person	5.71 (3.49–7.93)	4.30 (2.34–6.26)
Employment status: on sick leave	1.20 (-0.75–3.16)	1.42 (-0.82–3.65)
Compensated	1.09 (-0.99–3.16)	0.29 (-1.97–2.56)
Under dispute	-0.76 (-3.35–1.82)	1.01 (-1.68–3.71)
**Dependent = IEQ-J Perceived lack of empathy**	**(R**^**2**^ **= 0.02)**	
Age	0.01 (-0.04–0.05)	-0.01 (-0.05–0.03)
Sex: women	-0.03 (-0.22–1.15)	-0.10 (-1.20–1.00)
Duration: ≥ 1 year	1.62 (0.28–2.96)	1.50 (0.24–2.77)
Cause of injury: traffic accident	-0.67 (-1.91–0.58)	-0.11 (-1.21–1.00)
Liability for injury: another person	1.27 (-0.03–2.57)	0.69 (-0.40–1.78)
Employment status: on sick leave	-0.15 (-1.29–1.00)	-0.16 (-1.28–0.97)
Compensated	0.43 (-0.79–1.65)	0.45 (-0.67–1.57)
Under dispute	-0.26 (-1.78–1.26)	0.19 (-1.15–1.54)
**Dependent = PCS Total**	**(R**^**2**^ **= 0.18)**	
Age	0.03 (-0.17–0.25)	-0.06 (-0.25–0.14)
Sex: women	-0.60 (-6.05–4.84)	-2.25 (-7.78–3.29)
Duration: ≥ 1 year	10.41 (4.22–16.60)	9.40 (3.21–15.59)
Cause of injury: traffic accident	-0.98(-6.70–4.74)	0.97(-4.57–6.52)
Liability for injury: another person	5.19 (–0.79–11.18)	2.71 (–2.81–8.23)
Employment status: on sick leave	4.39 (-0.93–9.72)	4.04 (-1.57–9.64)
Compensated	6.30 (0.71–11.89)	7.20 (1.82–12.58)
Under dispute	-3.62 (-10.58–3.35)	-59 (-7.34–6.15)

Note: n = 71; IEQ-J, Japanese version of Injustice Experience Questionnaire; PCS, Pain Catastrophizing Score

## Discussion

The current study confirmed the validity and reliability of the IEQ-J. This study partially referred to consensus-based standards for the selection of health measurement instruments (COSMIN) checklist [29]. The COSMIN checklist indicates seven general requirements for evaluating methodological quality of studies on health-related patient-reported outcomes: internal consistency, reliability, measurement error, content validity, construct validity (subdivided into structural validity, hypotheses testing, and cross-cultural validity), criterion validity, and responsiveness [29]. The current study was highly qualified according to these requirements.

The factor structure derived from the current study differed from that of the original IEQ version. Three factors, *Severity/irreparability* (Items 2, 5, 6, 9, 10, and 11), *Blame/unfairness* (Items 3, 4, 7, and 12), and *Perceived lack of empathy* (Items 1 and 8), were proposed by EFA in the current study. This three-factor model indicated good fit to the data. However, *Perceived lack of empathy* is a distinctive factor of the IEQ-J. Thus, Item 4, “No one should have to live this way” belonged under *Severity/irreparability* in the previous study, but under *Blame/unfairness* in the current study. By contrast, Item 9, “Nothing will ever make up for all that I have gone through,” Item 10, “I feel as if I have been robbed of something very precious,” and Item 11, “I am troubled by fears that I may never achieve my dreams” belonged to *Blame/unfairness* in the previous study, but to *Severity/irreparability* in the current study. This result might be due to cross-cultural differences in meanings or interpretations when people encounter similar emotional situations. For example, Boiger et al. indicated that anger is condoned in the United States, but condemned in Japan; conversely, shame is condoned in Japan, but condemned in the United States [30]. The Japanese generally consider perceived lack of empathy as perceived injustice and indifference. They respect showing empathy to others without verbal communication, in order to harmonize social relationships. Furthermore, the Japanese are accustomed to perceiving empathy from others, and thus, once they perceive lack of empathy, it is interpreted as injustice and indifference.

In previous literature, forgiveness interventions, anger management interventions, and/or mindfulness meditation were considered useful for accident victims who perceived severe injustice [5]. The new factor, *Perceived lack of empathy* found in the current study might be a new target for treatment among Japanese patients who perceive severe injustice.

The correlation coefficient of IEQ-J and PCS total scores (r = 0.73, 95%CI: 0.59–0.82) was similar to that of IEQ and PCS total scores (r = 0.75 and *p* < 0.01) in Canadian patients with musculoskeletal conditions [1], and (r = 0.65, *p* < 0.001) in Spanish patients with fibromyalgia [13]; these showed high correlations.

We also investigated the association between demographic variables related to injury or pain and IEQ-J and PCS scores. Our study results followed past descriptions that victims whose injury occurred from another’s error or negligence are likely to perceive injustice [1,5]. We hypothesized that injury related to another’s error or negligence was associated with a higher IEQ-J score for *Blame/unfairness*; present findings support our hypothesis. Furthermore, the IEQ-J subscale scores for *Severity/irreparability* and *Perceived lack of empathy* increased when injury resulted from another’s error or negligence. A previous study reported that IEQ scores of subjects injured by motor vehicle accidents were significantly higher than those of subjects injured by work accidents [1]. In the current study, however, motor vehicle accident was not a dependent factor increasing IEQ-J scores. Liability for motor vehicle accidents was not described in the previous study, but many motor vehicle accidents might be caused by another’s error or negligence: This might be why IEQ-J scores of participants injured by motor vehicle accidents were increasing.

Although compensation and dispute are hypothesized as important factors for increasing IEQ-J scores, they did not contribute thusly in the current study. However, compensation significantly increased the IEQ-J subscale *Severity/irreparability* and PCS total scores. This result added new evidence to the academic field of perceived injustice.

In the current study, mean values of IEQ-J total scores 24.1 (SD 12.0, n = 71) tended to be lower than mean values among an Australian sample, 27.9 (SD 17.4, n = 163), as reported by Kennedy et al. [14]. They tended to be higher than those among a Canadian sample, 17.3 (SD 12.2, n = 150), as reported by Sullivan et al. [1]. The Australian sample was compensated, with an average disability duration of over four years, so these characteristics should increase IEQ total scores [14]. Although Sullivan et al. did not report duration of injury among the Canadian population, differences in each society’s compensation system might cause this discrepancy among Japanese, Australian, and Canadian populations [1,14]. According to factor structure, previous studies among Canadian [1], Spanish [13], and Australian [14] populations did not indicate cross-cultural differences, except for factor structure and error variances of Items 2 and 5 or Items 4 and 11 among the Australian population.

This study has some limitations. First, the sample is not necessarily representative of the Japanese population, even though the sample size is sufficient to confirm the validity and reliability of the IEQ-J. Second, we did not investigate socio-economic factors other than compensation (e.g., educational level, family structure, living area, and income) in the current study. Socioeconomic disparities may influence perceived injustice as well as common psychosocial factors [31].

## Conclusions

The current study provides evidence for the sound psychometric properties of the IEQ-J. Its three-factor model showed good fit to the data, even though its factor-structure differed from the original IEQ’s two-factor model. Pain duration of over a year and liability for injury by another statistically significantly increased IEQ-J total score.
